# Analyzing the Relationship Between Various Factors and Their Influence on the Success Rates of Meniscal Injury Procedures: A Prospective Cross-Sectional Study

**DOI:** 10.7759/cureus.76877

**Published:** 2025-01-03

**Authors:** Ramah Salah Mohammed Khalil, Ahmed Mohamed, Monzir Adam Ahmed Mohammed, Maysara Elsiddig, Abeer Eltahir Mohamed Ahmed Rabah, Reyad Abdallah, Hadeel M Sovla, Sara Hussein Mohamed Hussein

**Affiliations:** 1 Orthopedics and Trauma, Gezira Traumatology Center, Jeddah, SAU; 2 Trauma and Orthopedics, Gezira Traumatology Center, Wad Madani, SDN; 3 General Surgery, Al Aqiq General Hospital, Al Aqiq, SAU; 4 General Surgery, Burjeel Medical City, Abu Dhabi, ARE; 5 Orthopedics and Trauma, King Fahad Hospital, Al Bahah, SAU; 6 Surgery, Betsi Cadwaladr University Health Board, Wrexham, GBR; 7 Orthopedics, Gezira Traumatology Center, Wad Madani, SDN; 8 General Surgery, Wad Medani Teaching Hospital, Wad Madani, SDN

**Keywords:** age, anterior cruciate ligament (acl) injury, bmi, meniscal injury, postoperative outcomes

## Abstract

Background: Meniscal injuries pose a significant orthopedic challenge, demanding a thorough understanding of demographic and injury-related factors for successful treatment. The importance of age, body mass index (BMI), time from trauma to surgery, injury causes, patient occupation, and the presence of anterior cruciate ligament (ACL) injury in influencing postoperative outcomes prompted this prospective cross-sectional clinical study.

Methods: Conducted from November 2022 to July 2023 with 30 patients, this study utilized rigorous methodologies, employing both analysis of variance and Spearman's correlation analyses. The aim was to comprehensively assess the relationships among these factors and postoperative results in meniscal surgery patients.

Results: The study found that early surgical intervention significantly improved outcomes for meniscal injuries, with patients operated on within two months achieving 100% excellent and good results, compared to 50% for those treated after six months. Younger age, lower BMI, and sports-related injuries were associated with better postoperative Lysholm scores, while higher BMI and delayed surgery correlated with poorer outcomes. Patients with concurrent ACL injuries had a 95.4% rate of excellent and good results, compared to 50% for those with intact ACLs.

Conclusion: This study underscores the pivotal role of age, BMI, and ACL injury considerations in shaping effective treatment strategies for meniscal injuries. The nuanced insights gained from the analysis emphasize the critical importance of early surgical intervention, particularly in cases involving ACL injuries, to optimize postoperative outcomes.

## Introduction

The meniscus serves as a crucial intra-articular component, essential for preserving knee stability and mitigating the advancement of osteoarthritis (OA) [1]. Additionally, it plays a pivotal part in functions such as load distribution, absorbing shock, and the lubrication of the joint [2]. The menisci within the knee joints play a substantial role in facilitating joint lubrication, distributing weight, safeguarding cartilage, maintaining joint stability, and enhancing proprioception [3,4]. Consequently, an injury to the meniscus can disrupt these functions, leading to modifications in mechanical dynamics and biochemical processes that, when combined, set in motion a sequence of events culminating in the onset of posttraumatic OA [4]. Meniscus injuries frequently give rise to knee pain and functional limitations [5]. While conservative management is an option for addressing the symptoms of meniscus tears, surgical intervention is frequently pursued, making it one of the prevalent procedures in the field of orthopedics [6].

Surgical intervention is a common choice, often ranked among the recurrent orthopedic surgical procedures, while conservative approaches can address the symptoms of meniscal tears [6]. An arthroscopic partial meniscectomy (APM) is employed for removal when a meniscal tear is irreparable and leads to pain or a locking sensation. Nevertheless, more than 50% of patients undergoing partial meniscectomy may exhibit articular cartilage changes within six months after surgery, and subsequently, OA can develop within 10-20 years [4]. Additionally, managing patient expectations for surgical outcomes is challenging due to the variability in results. Meniscectomy aims to achieve three primary objectives: alleviating pain, restoring the individual’s preinjury level of daily activities, and preventing the onset of degenerative arthritis in the knee joint [7].

In cases of traumatic meniscus injuries, they are frequently concomitant with injuries such as anterior cruciate ligament (ACL) tears, medial collateral ligament tears, and chondral lesions, which can worsen the knee’s functionality. The primary focus in such situations should encompass both functional recovery and the enhancement of the quality of life, a factor that is gaining increasing significance but remains underexplored in the existing literature on this subject [8]. Approximately 30%-60% of ACL tears coincide with meniscal or chondral injuries during the initial reconstruction [9,10]. Among these, the lateral meniscus is the most frequently affected. This tendency can be attributed to the distinct structural and functional characteristics of the lateral meniscus, including its limited connections to the collateral ligament and popliteal hiatus, and increased mobility, which reduces the risk of ruptures and makes it less susceptible in a stable knee [11]. The menisci serve as secondary stabilizers in the knee joint, and it is widely recognized that the clinical outcomes of ACL reconstruction are notably compromised when there is a concomitant meniscal injury [8].

The advancement in diagnostic methods for identifying meniscal injuries has led to a substantial rise in arthroscopic procedures for treating such injuries in recent years. Initially, partial meniscectomy was a prevalent approach for patients who did not respond to nonoperative treatments [12]. The surgeons agree that meniscectomy could provide short-term pain relief and enhance knee function, whereas the long-term consequence of losing meniscal tissue is an acceleration in the early OA development [13]. In contrast to partial meniscectomy, meniscus repair procedures can reinstate its biomechanical function and hinder the progression of knee OA over the long term by preserving meniscal tissue. This has led to a consensus favoring the preservation of meniscal tissue whenever feasible [14]. Surgeons determine whether to proceed with meniscus repair based on two key criteria: location and severity of meniscal tear. Repair is considered when the tear is situated in the red-red zone or when sufficient meniscal tissue remains intact [15].

Repairing the meniscus in avascular regions poses a significant challenge to surgeons, potentially impeding the healing process of the injured meniscus and contributing to degenerative arthritis. Arthroscopic meniscal repair is an appealing, minimally invasive procedure associated with reduced complications and shorter hospital stays. The study aims to identify the factors that play a role in determining the success of this procedure. This study’s significance lies in its potential to enhance our understanding of meniscal repair in avascular regions, leading to improved outcomes and patient care.

## Materials and methods

A prospective cross-sectional clinical study was performed in Sudan from November 2022 to July 2023, involving 30 patients who underwent meniscal repair using the arthroscopic outside-in technique.

The study included patients diagnosed with meniscal tears amenable to arthroscopic outside-in repair based on MRI and clinical examination. The inclusion criteria specified an age range of 18-50 years, a stable or stabilized knee joint (with no major ligament instability requiring concurrent repair), and the absence of significant comorbidities that could affect surgical or rehabilitation outcomes. All arthroscopies were systematically performed utilizing nonabsorbable suture materials, including Ethibond® (Ethicon, Inc., Somerville, NJ) and FiberWire® (Arthrex Inc., Naples, FL). To maintain consistency and reduce variability, one single surgeon conducted all surgical procedures during the study.

Patients were excluded if they had a history of prior surgical interventions on the affected knee, such as meniscectomy or ligament reconstruction, advanced OA or severe cartilage damage, or complex, degenerative, or irreparable meniscal tears. Other exclusion criteria included systemic diseases that impede wound healing, such as diabetes mellitus (hemoglobin A1c >7%), autoimmune disorders, or chronic steroid use. Patients who were unable to participate in physiotherapy due to physical or logistical limitations, as well as those with acute or chronic infections, including septic arthritis, were also excluded. Additional exclusionary factors include failure to attend follow-up appointments or exhibit poor compliance.

After surgery, patients engaged in a systematic rehabilitation program aimed at enhancing recovery. This program comprised physiotherapy sessions to improve strength, flexibility, and range of motion. Weight-bearing activities were gradually introduced to facilitate healing while reducing the likelihood of reinjury.

The researcher conducted the data collection process, ensuring it was well-informed and requiring no further investigations. Patients were subsequently followed up for three months. The collected data were entered, coded, and analyzed using the Statistical Package for the Social Sciences, version 27 (IBM Corp., Armonk, NY). Descriptive statistics were conducted to analyze the demographic characteristics, including age, gender, and other relevant variables, of included patients. We performed cross-tabulation to analyze the categorical relationship between demographics and Lysholm score. An analysis of variance (ANOVA) test was conducted to signify this relationship. Finally, the Spearman correlation test was conducted to investigate the strength of the relationship between demographic factors and the success of procedures for meniscal injuries based on the Lysholm score.

The study took several steps to ensure the ethical consideration of recruited patients. The study was conducted after obtaining permission from the hospital administration authority to ensure compliance with ethical standards. In addition, informed consent was obtained from each patient participating in the study. To safeguard patient confidentiality, records were assigned serial numbers, and all participating patients provided verbal consent, indicating their willingness to enroll in the study.

## Results

Table 1 presents the demographic details of all patients. Out of the 30 enrolled patients, the majority were male, constituting 96.7% (n = 29). The patients’ age distribution was as follows: nine patients (30%) aged 18-23 years, 14 patients (46.7%) aged 24-27 years, four patients (13.3%) aged 28-31 years, and three patients (10%) aged 32 years and above. Patients were categorized based on body mass index (BMI) into three groups: eight patients (26.7%) with a BMI <25, 18 patients (60%) within the BMI range of 25-30, and four patients (13.3%) with a BMI >30.

**Table 1:** Demographic details of patients BMI: body mass index; ACL: anterior cruciate ligament; RTA: road traffic accident

Demographics, injury characteristics, and postoperative outcomes		Frequency	Percentage
Gender	Female	1	3.2
	Male	29	93.5
	Total	30	100.0
Age	18-23	9	29.0
	24-27	14	45.2
	28-31	4	12.9
	More than 31	3	9.7
	Total	30	96.8
BMI	25-30	18	58.1
	Less than 25	8	25.8
	More than 30	4	12.9
Side of injury	Left	13	41.9
	Right	17	54.8
	Total	30	96.8
The period since trauma to injury	0-2 months	10	32.3
	2-4 months	6	19.4
	4-6 months	6	19.4
	More than six months	8	25.8
Causes of meniscal injury	Sports	17	54.8
	RTA	5	16.1
	Fall down	8	25.8
Occupation	Nonathlete	21	67.7
	Athlete	9	29.0
Complications of meniscal repairs	Yes	28	90.3
	No	2	6.5
ACL injury	Yes	22	71.0
	No	8	25.8
Postoperative Lysholm score	65-83	5	16.1
	84-90	17	54.8
	91-100	8	25.8

Patients were classified according to the side of injury, with 17 patients (56.7%) experiencing injuries on the right side and 13 patients (43.3%) on the left side. As for the injury site, 16 patients (53.3%) had medial injuries, and 14 patients (46.7%) had lateral injuries. The period from trauma to surgery was categorized into four groups: zero to two months (10 patients, 33.3%), two to four months (six patients, 20%), four to six months (six patients, 20%), and more than six months (eight patients, 26.7%). The leading causes of meniscal injuries were sports-related incidents, accounting for 60% of cases, followed by falls (23.3%) and road traffic accidents (RTAs) (16.7%). Patients were categorized as athletes (30%) and nonathletes (70%). In terms of complications, 6.7% of cases were reported to have experienced issues, while the majority of patients (93.3%) did not encounter any complications. Furthermore, it was noted that, in the majority of meniscus injury cases, an associated ACL injury was observed in 73.3% of patients, while 26.7% had an intact ACL.

The study unveiled key findings on patient characteristics and postoperative outcomes (Table 2). Age played a significant role, as all patients (100%) in the 18-23 age group achieved excellent and good outcomes. In the 24-27 age group, 92.8% also achieved excellent and good outcomes, while in the 28-31 age group, 75% of patients experienced these positive results. Notably, no patients in the age group over 30 achieved an excellent outcome. Another crucial factor was BMI, as all patients (100%) in the group with a BMI less than 25 attained excellent and good scores.

**Table 2:** Cross-tabulation between demographics and Lysholm score BMI: body mass index; RTA: road traffic accident; ACL: anterior cruciate ligament

Patient characteristics		Postoperative Lysholm score			Total
		65-83	84-90	91-100	
Age	18-23	5	4	0	9
	24-27	0	13	1	14
	28-31	0	0	4	4
	More than 31	0	0	3	3
BMI	25-30	5	13	0	18
	Less than 25	0	4	4	8
	More than 30	0	0	4	4
The period since trauma to injury	0-2	5	5	0	10
	2-4	0	6	0	6
	4-6	0	6	0	6
	More than six months	0	0	8	8
Causes of meniscal injury	Sports	5	12	0	17
	RTA	0	4	1	5
	Fall down	0	1	7	8
Occupation	Nonathlete	5	16	0	21
	Athlete	0	1	8	9
ACL injury	Yes	5	17	0	22
	No	0	0	8	8

However, in the group with a BMI of 25-30, 83.3% had excellent and good scores, while in the group with a BMI over 30, only 50% achieved excellent and good scores. The time elapsed between trauma and surgery was found to influence outcomes. Patients who underwent surgery within zero to two months had a 100% rate of excellent and good outcomes. In group 2 (two to four months), all patients achieved excellent and good outcomes. In group 3 (four to six months), 83.3% of patients had excellent and good outcomes, while in group 4 (more than six months), 50% showed excellent and good outcomes. The mode of trauma also correlated with outcomes. Sports-related injuries led to excellent and good outcomes in 94.4% of the patients, while RTAs resulted in 80% achieving these outcomes. Falls accounted for excellent outcomes in 57.1% of patients. Patients’ professions were associated with outcomes; nonathlete patients had 80.9% excellent and good outcomes, while athlete patients achieved these outcomes in 88.8% of cases. Furthermore, the presence of an ACL injury played a significant role, with patients having an ACL injury (95.4%) achieving excellent and good outcomes, while patients with an intact ACL (50%) had good outcomes.

Table 3 presents the results of an ANOVA examining the relationship between various demographic factors and the Lysholm score, which is a measure of postoperative outcomes. The ANOVA results highlight the significant impact of several demographic factors on postoperative Lysholm scores. Age, BMI, the time between trauma and surgery, causes of injury, occupation, and the presence of an ACL injury all showed highly significant associations (p < 0.001) with postoperative outcomes.

**Table 3:** Relationship between demographics and Lysholm score (ANOVA) ANOVA: analysis of variance; BMI: body mass index; ACL: anterior cruciate ligament

ANOVA (between groups)	Mean square	F	Sigma
Age	9.204	37.889	0.000
BMI	5.204	27.774	0.000
The period since trauma to injury	16.129	39.803	0.000
Causes of meniscal injury	7.771	31.051	0.000
Occupation	2.679	76.866	0.000
ACL injury	2.933	-	-

In Table 4, Spearman’s correlation analysis, demonstrating highly significant p values (p < 0.01), unveils several important relationships among key variables. Age demonstrates a strong positive correlation with BMI (ρ = 0.773, p < 0.01), the duration between trauma and surgery (ρ = 0.913, p < 0.01), causes of meniscal injury (ρ = 0.749, p < 0.01), patient occupation (ρ = 0.744, p < 0.01), and the presence of an ACL injury (ρ = 0.761, p < 0.01). Notably, age also exhibits a robust positive correlation with postoperative Lysholm scores (ρ = 0.854, p < 0.01), suggesting that younger patients tend to achieve better postoperative outcomes.

**Table 4:** Correlation analysis **Highly significant p values (p < 0.01) BMI: body mass index; ACL: anterior cruciate ligament

Spearman’s rho	Age	BMI	The period since trauma to injury	Causes of meniscal injury	Occupation	ACL injury	Postoperative Lysholm score
Age	1.000	0.773^**^	0.913^**^	0.749^**^	0.744^**^	0.761^**^	0.854^**^
BMI	0.773^**^	1.000	0.854^**^	0.915^**^	0.837^**^	0.797^**^	0.759^**^
The period since trauma to injury	0.913^**^	0.854^**^	1.000	0.887^**^	0.802^**^	0.795^**^	0.854^**^
Causes of meniscal injury	0.749^**^	0.915^**^	0.887^**^	1.000	0.872^**^	0.796^**^	0.770^**^
Occupation	0.744^**^	0.837^**^	0.802^**^	0.872^**^	1.000	0.921^**^	0.815^**^
ACL injury	0.761^**^	0.797^**^	0.795^**^	0.796^**^	0.921^**^	1.000	0.859^**^
Postoperative Lysholm score	0.854^**^	0.759^**^	0.854^**^	0.770^**^	0.815^**^	0.859^**^	1.000

Conversely, BMI shows significant positive correlations with age (ρ = 0.773, p < 0.01), duration between trauma and surgery (ρ = 0.854, p < 0.01), causes of meniscal injury (ρ = 0.915, p < 0.01), patient occupation (ρ = 0.837, p < 0.01), and the presence of an ACL injury (ρ = 0.797, p < 0.01). However, it demonstrates a slightly negative correlation with postoperative Lysholm scores (ρ = -0.759, p < 0.01), indicating that a higher BMI is associated with slightly poorer postoperative scores.

Furthermore, duration between trauma and surgery is positively correlated with causes of meniscal injury (ρ = 0.887, p < 0.01), patient occupation (ρ = 0.802, p < 0.01), and the presence of an ACL injury (ρ = 0.795, p < 0.01), suggesting that a longer duration between trauma and surgery may be associated with specific injury patterns and patient conditions. Causes of meniscal injury are positively correlated with patient occupation (ρ = 0.872, p < 0.01) and the presence of an ACL injury (ρ = 0.796, p < 0.01).

The postoperative Lysholm scores exhibit a significant positive correlation with age (ρ = 0.854, p < 0.01), indicating that younger patients tend to achieve better postoperative outcomes.

These correlations provide insights into the complex interplay of demographic and injury-related factors with postoperative outcomes following meniscal surgery, suggesting that age, BMI, the duration between trauma and surgery, the causes of injury, patient occupation, and the presence of an ACL injury are all significant factors that may influence the recovery and outcomes of patients who undergo meniscal surgery. These findings are valuable for healthcare professionals in understanding and managing the factors that contribute to postoperative success or challenges in meniscal injury cases.

## Discussion

The present study identified a significant correlation between BMI and postoperative outcomes following meniscal surgery. Notably, patients with a BMI <25 demonstrated a remarkable 100% rate of good and excellent outcomes compared to those in the BMI range of 25-30, where the rate was 77.7%. This observation suggests that individuals with lower BMI scores tend to achieve more favorable postoperative results. The rationale behind this trend is that individuals with lower BMI values often possess lighter and healthier body compositions, making them more agile and potentially better suited for a smoother recovery process.

In the present study, patients in the age groups of 18-23 years and 24-27 years achieved excellent and good outcomes. However, a previous study highlighted that patient age (≥50 years) was a significant factor associated with clinical failure [4]. While the present study did not predominantly include patients older than 50 years, Hong et al. [4] clarified that older age is a barrier to achieving positive postoperative results following meniscal surgery.

In a previous study, meniscal tears were linked with differences in patient improvement, particularly between traumatic and degenerative tears [5]. Similarly, the present study investigated whether the nature of meniscal tears had a significant impact on clinical outcomes, which allowed differentiating between patients with traumatic and degenerative tears. This holds significant importance in explaining whether those with traumatic tears exhibited more favorable results in postoperative improvement compared to patients with degenerative tears. This analysis sheds light on the potential influence of tear type on clinical outcomes, offering insights that may affect the functional outcomes following meniscal surgery.

Previous studies have reported that APM performed to correct degenerative tears yielded outcomes similar to physical therapy and sham surgery, suggesting a significant role of underlying OA in patient symptoms [16-18]. One of the previous studies found that 84.5% of patients with traumatic meniscal tears had concurrent ACL ruptures [1], which was consistent with other research [19]. This result highlights the strong association between ACL injury and the prevalence of meniscal repair, suggesting that ACL injury is a crucial factor influencing the need for meniscal repair [19-21]. The present study examined various demographic and injury-related factors and their impact on postoperative outcomes following meniscal surgery. Notably, age exhibited a robust positive correlation with postoperative Lysholm scores. However, higher BMI showed a slight negative correlation with postoperative scores, indicating that elevated BMI may be associated with slightly lower postoperative outcomes.

Several studies have documented superior healing rates among patients who undergo concurrent ACL reconstruction alongside meniscus surgery [19-21]. This phenomenon is attributed to the release of intra-articular growth factors from bleeding tunnels during ACL reconstruction, creating an optimal environment for meniscus healing [22,23]. Many meniscal tears accompanied by ACL injuries are often associated with sports-related activities, which are more prevalent among young patients. These factors collectively contribute to the increased prevalence of meniscus repair in patients with concurrent ACL injuries. The present study explored a wider range of factors influencing postoperative outcomes following meniscal surgery, offering valuable insights for healthcare professionals managing patients with meniscal injuries.

The finding of the present study suggested that while high BMI is not significantly associated with repair surgery failure, high body weight might be a more relevant factor to consider when planning meniscus repair surgery. The reasoning behind this could be the increased joint stress associated with greater body weight, which may lead to more severe meniscus injuries. In contrast, a meta-analysis indicated that BMI does not significantly affect repair surgery failure rates [24]. Furthermore, another study found an inverse correlation between BMI and International Knee Documentation Committee and Tegner activity level scores, suggesting that BMI alone may not be a strong contraindication for meniscus repair surgery [25]. Importantly, BMI can also be influenced by factors like a patient’s height.

Partial meniscectomy or meniscus repair is typically considered when conservative treatments fail to alleviate symptoms such as pain and knee locking, particularly within a two-week timeframe. Majeed et al. [19] also reported that repairing the meniscus within six weeks of injury is associated with a lower failure rate, especially in patients with concomitant ACL injuries. The present study findings are aligned with the idea that delaying arthroscopic surgery, especially in cases of ACL deficiency, reduces the chance of meniscal repair. This is likely because the meniscus plays an essential role in regaining knee functionality, and postponing surgery can lead to the progression of meniscus tears, especially in ACL-deficient conditions [26]. It is presumed that the extent of meniscus damage can influence surgeons’ decisions on whether to pursue meniscal repair due to concerns about its limited healing potential.

The study findings emphasized the importance of considering factors like age, BMI, the duration between trauma and surgery, causes of injury, patient occupation, and the presence of an ACL injury when evaluating and predicting patient recovery after meniscal surgery. The findings provide valuable insights into the complex association of demographic and injury-related factors that impact postoperative success or challenges in meniscal injury cases. These results provide important insights for healthcare professionals, aiding their comprehension and handling of factors influencing postoperative outcomes or difficulties in cases of meniscal injuries.

Several limitations in this study should be considered. These limitations include a relatively small sample size of 30 patients and a short follow-up period of only three months. Furthermore, the evaluation in the study is primarily clinical, lacking the inclusion of MRI follow-up and arthroscopic second-look assessments. This omission means that asymptomatic failures may not have been detected. Another limitation is the absence of data regarding specific patient habits, such as smoking, and how these habits may influence the healing of repaired menisci. This missing information may have an impact on the overall understanding of the factors affecting meniscal repair outcomes. Finally, the study did not include observations of patients from older age groups, which could be a limitation in assessing the full spectrum of age-related outcomes in meniscal surgery.

For future research, investigating the mechanisms underlying the observed correlations and investigating potential factors that mediate these relationships is crucial. Further studies could explore the specific biological and biomechanical processes that link demographic factors to postoperative outcomes. Longitudinal studies with large and diverse samples may further clarify the understanding of the relationships identified in this study, further enhancing the management of meniscal injuries and the prevention of secondary complications like OA.

## Conclusions

The present study showed the complex correlation between demographic and injury-related factors and postoperative outcomes in meniscal surgery. The patient's age, BMI, the duration between trauma and surgery, causes of injury, occupation, and the presence of an ACL injury significantly impact postoperative Lysholm scores. Notably, age emerged as a strong predictor of favorable postoperative outcomes, while higher BMI was associated with slightly lower scores. These findings shed light on the multifaceted nature of factors influencing patient recovery following meniscal surgery, providing valuable insights for healthcare professionals seeking to enhance patient care and improve outcomes.
